# New Hygienic Hospital Fittings

**Published:** 1907-04-06

**Authors:** 


### NEW HYGIENIC HOSPITAL FITTINGS.

### DOULTON'S PATENT REVOLVING HOSPITAL BATH.

This bath has been specially designed to meet the suggestions of Dr. Mackintosh, Medical Superintendent Western Infirmary, Glasgow, and has been fitted up in the new pavilion recently added to that institution. This bath affords facilities for the thorough cleansing of every part of the bathroom, and at the same time, effects great economy of space. The bath is made of cast iron, porcelain enamelled inside and out, with a roll edge, and is recessed at the top for a standing waste. It is much lighter and less costly than an earthenware bath, and has all its advantages. The waste is made extra large and discharges into a gun-metal trap bolted to the floor and secured by means of a packed joint. The bath can be moved about on this swivel joint. In this way the whole of the floor and walls can be kept thoroughly cleansed, at the same time allowing a nurse or assistant to get on either side of the bath. At the lower end are two rubber tyred wheels with a brake, so that the bath revolves with the greatest ease without putting the slightest strain on the swivel joint, and by means of the brake it can be fixed at any part of the floor. In hospital construction economy of space is always important, and in a hospital bathroom it is essential that the nurse should be able to get at either side of the bath. By using the revolving bath, which is illustrated on our advertisement pages, this is accomplished in a simple and thoroughly efficient way. The water is supplied through a special non-scalding valve fixed on the wall, with a removable key, and is so arranged that cold, tepid, or hot water can be obtained as required, the cold water always coming first so that there is no possible chance of scalding.

